# Supplementary material for: MRGPRX2-expressing mast cells are increased in the GI tract of individuals with active inflammatory bowel disease and hereditary α-tryptasemia
Source: Front Allergy. 2026 Jan 21;6:1726096. doi: 10.3389/falgy.2025.1726096 (PMC12868144; doi:10.3389/falgy.2025.1726096)

**Supplemental Table 1a.** Demographics of HαT Participants (N=8)

| Sample number | Age at collection | Sex at birth/race/  ethnicity | Diagnosis and TPSAB1 CNV | MC per HPF in small intestine | symptom onset | KIT | Serum tryptase | History of Anaphylaxis (Y/N) | Analysis |
| --- | --- | --- | --- | --- | --- | --- | --- | --- | --- |
| 5 | 50 | F/white/Caucasian | HAT 2:3 | 80 | Adult | Negative | 14.5 | Yes | C |
| 6 | 25 | M/white/Caucasian | HAT 3:2 | 32 | Adult | Negative | 17 | No | C |
| 7 | 67 | F/white/Caucasian | HAT 3:2 | 35 | Adult | Negative | 15.5 | Yes | C |
| 17 | 40 | M/white/Caucasian | HAT 2:3 | 75 | Adult | Negative | 12.8 | No | C |
| 18 | 55 | F/white/Caucasian | HAT 3:2 | 48 | Adult | Negative | 17 | Yes | C |
| 19 | 33 | M/white/Caucasian | HAT 2:3 | 35 | Pediatric | Negative | 10.7 | No | C |
| 20 | 31 | F/white/Caucasian | HAT 3:2 | 62 | Adult | Negative | 11.6 | yes | C |
| 21 | 31 | F/white/Caucasian | HAT 3:2 | 34 | Pediatric | negative | 10.6 | yes | C |

**Table 1b.** Demographics of Control Participants (N=4)

| Sample number | Age at collection | Sex at birth/race/ethnicity | TPSAB1 CNV | Serum Tryptase | analysis |
| --- | --- | --- | --- | --- | --- |
| 22 | 44 | F/white/Caucasian | 1:3 | N/A | C |
| 23 | 49 | F/white/Caucasian |  | 5 | C |
| 24 | 23 | F/white/Caucasian | 0:4 | 4.1 | C |
| 25 | 42 | F/white/Caucasian | 0:4 | 4.7 | C |

**Supplemental table 2.** Mass cytometry panel, clones, and sources.

**Supplemental table 3.** R Packages used in transcriptomics analysis

| **Package** | **Version** | **Source** |
| --- | --- | --- |
| **R** | 2023.06.1+524 | CRAN |
| **Seurat** | v5.3.0 | CRAN |
| **SCTransform (in Seurat)** | 0.4.2 | CRAN |
| **ggplot2** | 3.5.2 | CRAN |
| **cowplot** | 1.1.3 | CRAN |
| **Matrix** | 1.6-4 | CRAN |
| **stringr** | 1.5.1 | CRAN |
| **DESeq2** | 1.42.1 | Bioconductor |

**Supplemental Figure 1.** CyTOF gating strategy


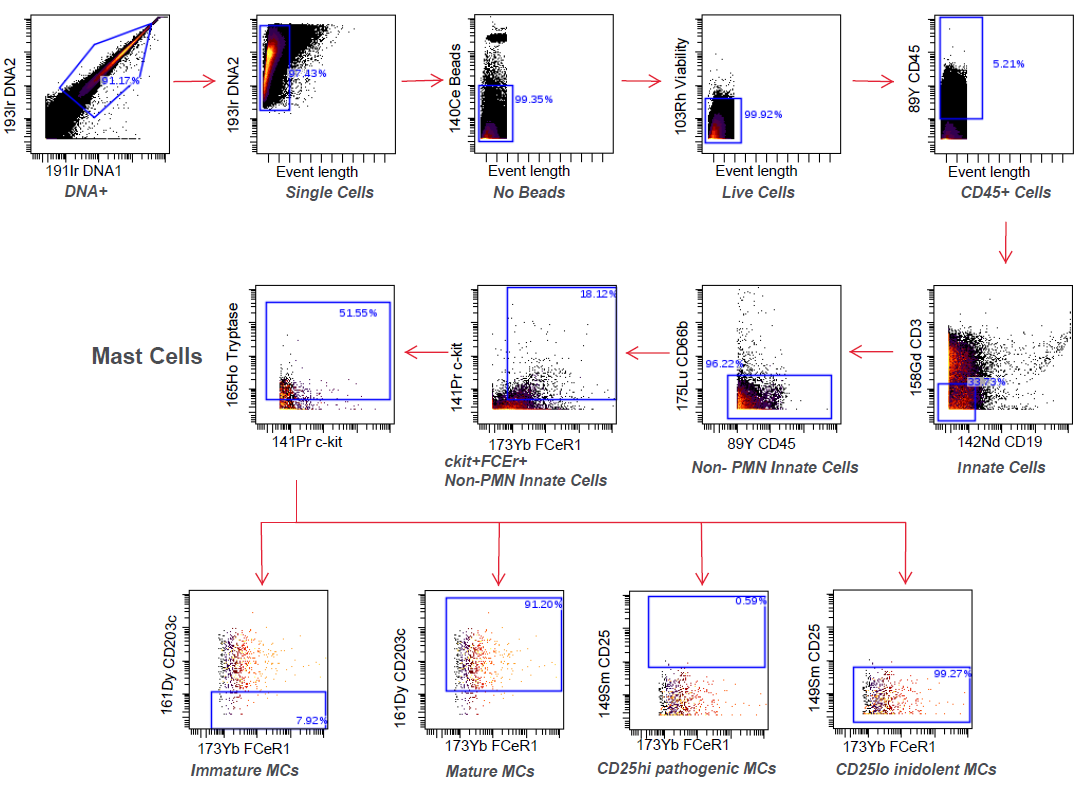

Supplement: Supplementary file 1 [file Datasheet1.docx]
